# Behaviour settings as a way to order types of situations for the study of speech aids

**DOI:** 10.1098/rstb.2023.0290

**Published:** 2024-08-07

**Authors:** Konrad Zieliński, Joanna Rączaszek-Leonardi

**Affiliations:** ^1^ Human Interactivity and Language Lab, Faculty of Psychology, University of Warsaw, 00-183 Warsaw, Poland

**Keywords:** behaviour settings, ecological psychology, enactive approach, speech aids, conversational analysis, laryngectomy

## Abstract

This article revisits the notion of behaviour settings, coined by Roger G. Barker (Barker 1968, *Ecol. Psychol*. **28**, 39–55 (10.1080/10407413.2016.1121744)), as a useful concept for the analysis of situations and communicative needs of persons after larynx removal surgery (laryngectomy). We claim that behaviour settings offer a way to characterize types of situations and types of participation, which, in turn, helps to identify aspects of communication where compensation is needed; these steps are crucial in the design process of reliable and context-sensitive speech aids. Moreover, we advocate complementing the behaviour setting concept as a unit of analysis with modern developments in the cognitive sciences, such as conversational analysis of co-operative actions (Goodwin 2017, *Co-operative action (learning in doing: social, cognitive and computational perspectives)*. Cambridge: Cambridge University Press (10.1017/9781139016735)) and the analysis of multi-perspectival experience (De Jaegher 2021, *Phenomenol. Cogn. Sci*. **20**, 847–870 (10.1007/s11097-019-09634-5)). Such an integration of macro- and micro-level patterns should help discover the relevant relations and values in particular situations. We illustrate our claims with examples from Barker’s own work and from our ongoing analyses of the everyday life of persons after laryngectomy.

This article is part of the theme issue ‘People, places, things, and communities: expanding behaviour settings theory in the twenty-first century’.

## 1. Introduction

The psychology of human behaviour in its real ecological context has been Roger G. Barker’s main explanatory target and the subject of research conducted by the group that he led. The group dedicated their work to cataloguing the everyday life of inhabitants of Oskaloosa, a small town in Kansas. During over 25 years of operation, from 1947 to 1973, the Midwest Psychological Field Station had been documenting the lives of 800 inhabitants, especially children [1]. They argued that psychology, in order to be useful for society, should account for the kinds and distributions of situations in nature, and that this knowledge should complement the experimental laboratory work which was dominating this field of science at the time.

Barker’s group laid down foundations—concepts, methodologies, research practices—for observing human behaviour in truly ecological, naturalistic settings. They proposed the concept of behaviour settings understood as immediate environments of human behaviour and experience, with designated boundaries in space, time and other criteria pertaining to the people populating the setting [2]. This conceptualization was motivated by the discovery that situations and places are at least equally good predictors of people’s behaviour as individual features; for example, people in the post office or in the bank behave similarly to people in other facilities serving similar purposes in a similar part of the day. Tools were designed to individuate particular settings, based on the spatial and behavioural distinctness from other settings. Focusing predominantly on children, the group documented how people operate in mundane everyday activities, and in particular places, day times and routines, with the main aim to discover and document the systematicities of behaviours across people within a setting and the frequency of encountered settings by different individuals.

We turned to Barker’s research and methodology (not widely used or known in the world of mainstream experimental psychology) because of a specific research need. For the last 6 years, our research group has been analysing the communication perturbed by larynx removal surgery. This surgery limits possibilities for natural verbal interaction, forcing the affected person to rely on substitutive voice generation mechanisms and compensatory techniques [3]. While studying speech aid designs, we were surprised at the low versatility of the solutions proposed: the current technological research on new speech aids is mainly driven by the ‘clarity of signal transmission’ goal, while speech intelligibility and naturalness is measured in listening tests performed in an acoustic laboratory setting—only one of the various possible settings and entirely non-typical.

What seemed to be missing is the consideration that communication occurs in multiple environments and multiple interpersonal contexts, each with its specific requirements for voice quality beyond simple ‘intelligibility’. People need to be quiet and tender, sometimes blend with the acoustic environment and have voice dynamics adapted to a particular situation. This variety of complex and nuanced needs of a person using a device in interaction with others remains unidentified. Their identification requires the study of particular interactions in their real settings, with a proper methodology and a humane, qualitative reflection, as well as a framework that could help recognize the classes of contexts and their specific properties and requirements for voice characteristics.

**Figure 1.**
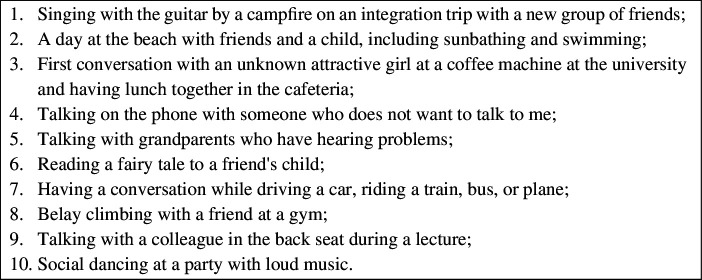
List of situations that were problematic for one of the authors who had received a laryngectomy.

The essential context of this work is our first-person and second-person experience with perturbed communication. Our research team is led by a researcher who underwent laryngectomy; therefore, we have the perspective of both interaction observers and participants. In the early period after the surgery, Konrad compiled a list of examples of particular interactions that felt problematic or frustrating, such as encountering a stranger, reading a story to a child or talking while driving a car (for other examples, see figure 1).

We further conducted qualitative ethnomethodological studies with a group of nine laryngectomees with varying professional backgrounds and from various areas of Poland, resulting in more examples of such problematic situated experiences [4]. What was needed was a tool for careful recognition of whether these individual situations could be clustered into groups, possibly requiring different technological (or interactive) assistance to restore the desired engagement and agency. Such *types of engagement with others* within types of situations would, we felt, be more informative for the analysis and formulation of assistive technology design requirements than unique examples of particular interactions.

Barker’s behaviour settings concept seemed to be suited to our aims, as one of the very few notions in psychology encompassing human activity and environmental features at the same time. Barker’s assumption that the stream of human behaviour could be divided into specific units with designated boundaries was crucial for that purpose. Identifying relevant behaviour settings would allow us to identify relevant communicative situations in a stream of everyday activities together with relevant structures of the environment that comprised the settings, providing guidelines for assistive technology.

However, we noticed that the generality of Barker’s approach and the ‘objectivity’ he strived for, while required for the recognition of such specific units, might not be sufficient to provide an understanding of *how* a disability might compromise participation in those situations. Barker’s approach is valuable to document the difference in frequency of participation in various settings, e.g. by laryngectomees and other groups, but the specific reasons for such differences would not be clear. These require understanding the specific relations among the participants in a specific setting and, ideally, some access to their experiences. Studying these ‘microscale’ factors requires a perspective which could help recognize the specific social roles and types of engagement as well as the values and types of agency required by the situation, the realization of which might be perceived as compromised by participants in a particular setting.

Therefore, besides illustrating the utility of Barker’s work for the above research project, an additional theoretical contribution of this paper is to consider the behaviour settings theory in the context of developments in linguistics, anthropology and cognitive science, concerning methods for studying human interaction and experience, such as engaged epistemology and conversational analysis (CA). We claim that such methods, even if they would probably not have been embraced by Barker at the time of his framework’s creation, allow for a deeper understanding of a behaviour setting which—for some purposes, such as ours—seems indispensable. These approaches foreground the engagements of participants in a variety of personal relations, recognize a variety of experiences [5], and allow for the microanalysis of movements and intonations in particular moments to be informative about these engagements [6]. Enriching Barker’s framework with such dimensions and timescales of analysis would allow us to understand the settings not only in terms of their frequency, frequency of actions within the settings, and spatial and temporal properties, but also in terms of values, which are realized jointly in co-actions [7] revealed by both by patterns of movement in interactions, as well as by first-person experiences and feelings that appear in a setting.

Let us underscore that these elaborations do not undermine Barker’s framework: it is one thing to recognize a standing behaviour pattern replicated by society and sustained by human activity, and it is another to know how people realize their values and feel while participating in such a pattern. By combining those approaches, we aim to both distinguish the relevant settings and to understand what people within these settings need for their participation to be agentive where needed, so that the setting is inclusive, and all participants’ roles can be fulfilled. We think that without compromising Barker’s focus on the overall structures of human–environment engagement, his method can be complemented by the perspectival analyses of experience and micro-analyses of movement in interactions. These complementary approaches could inform each other about the possibilities to transform participation through the use of technology and to transform settings into more inclusive environments. At the same time, Barkerian objectivity is very much needed to delimit the sensible units of analysis in order to systematize the qualitative research within the proposed theoretical enrichments.

The structure of the paper is as follows: in the next section, we introduce Barker’s behaviour setting concept and its usefulness as a starting point to catalogue the frequency and structure of situations encountered by laryngectomees. Section 3 presents the possible complementary approaches which enrich the knowledge about the settings in terms of the kinds of interactions, patterns of participation and experiences of the participants. Section 4 provides examples of the advantages of such enrichment based on Barker’s own work and our own analyses. In §5, we call for a research pipeline encompassing the above elements, based on the enriched approach of Barker, which could be the beginning of a more comprehensive and individualized methodology for assistive technology design.

## 2. The methodology of Barker’s ecobehavioural science

Barker’s research was motivated by a seemingly mundane observation that among the best predictors of individual action at a particular time is the ‘setting’ or a ‘situation’ one finds oneself in. In the wake of the World War II tragedy, the effort of many researchers in social sciences was directed towards discovering what motivates particular behaviours of particular people at a particular time and place and how development instils adaptive and maladaptive patterns of behaviour. While much philosophical, sociological and psychological work was directed at discovering the individual cognitive, personality and motivational characteristics or particular trajectories of early socialization, ecological psychologists pointed out that the structure of the environment holds vital cues for behaviour, development and education that are often overlooked.

Following Kurt Lewin, psychologists called for a ‘psychological ecology’ [8]. One of the most advanced realizations of this postulate, performed by a research group led by Roger Barker, hinged on the concept of behaviour settings, extraindividual units with designated boundaries in space and time, in terms of which it is possible to describe the immediate environments of human behaviour and experience [2]. Acknowledging the importance of such settings for understanding people’s behaviour, their functioning, and their well-being in the world, required novel methods to identify, count and study them. Barker’s project aimed at developing such methods.

According to Barker, ‘A behavior setting consists of one or more standing patterns of behavior-and-milieu’ [2, p. 19]. It is treated as a unit of analysis and is characterized by a relative coherence and distinctness from other units, in which behaviours are intertwined with a particularly structured environment: ‘The behavior patterns of a behavior setting are attached to particular constellations of non-behavioral phenomena’, within ‘univocal temporal–spatial coordinates. Such as a basketball game, or a class lesson’. Identifying behaviour settings is of primary importance for a psychologist, because they are ‘stable, extraindividual units with great coercive power over the behavior that occurs within them’ [2, p. 18]. Defining such extraindividual units allows the enumeration of likely possibilities for actions of an individual in a particular setting, characterizing their ambience or context [8].

We will follow the details of Barker’s methodology of data gathering as exposed in one of his most prominent early works co-authored with Wright, *One boy’s day* (1951), a ‘remarkable scientific document’ aiming to describe in detail the functioning of a person during the whole day [9]. The 428-page book consists of observations of one day in the life of Raymond, a 7-year-old boy. A very strict method leads to the creation of a record which characterizes the settings in which the boy participates, enables him to count the frequency of actions within the settings, and through this characterizes the particular pattern of activity and involvements of an individual, which can then be compared with other individuals. In Barker’s words, researchers create a ‘specimen of the behaviour and of the cultural and psychological habitat of a child’. Barker describes the ensuing report as both an objective record (actions of a boy that could be registered by the skilled observers) and an interpretive record (what observers ‘inferred as to the meanings to the boy of his behaviour and of the persons, things, and events that he saw and heard and felt throughout the day’ [9, p. 1]). Presumably, differences in renderings of the psychological ecologies of different individuals would be as informative about the possible differences in their behaviour as (or, in some cases, more so than) knowing the individual personal characteristics.

Let us focus closely on the method as described in *One boy’s day*. Please note that we are focusing here on the method of data gathering, not on the later work on the behaviour settings identification (described in detail in [10]). We render it here in detail in order to present the method, but also to see what the researchers included in the records and in what manner, what they omitted on purpose (and whether they did so successfully) and what was possibly unintentionally neglected in the record.

### (a) Relation observer–observed

Barker was aware that reporting on behaviour is mediated by ‘a number of operations’, each with a probability of error. Records are made by ‘watching the behavior and reporting it in words’ [9, p. 4]. The main problem was how to keep the situation natural and observe naturally occurring behaviour, when the presence of an observer ‘changes the situation and hence the actions and feelings of the person observed’ [9, p. 5]. The answer hinges on applying several guidelines: (i) make the interference minimal, (ii) define it and (iii) hold it constant.

In order to minimize the dominance of the record by the point of view of a single observer, several (eight) researchers were employed to perform the task, and their relation and attitude towards the observed were presented as follows: seven of the eight observers were familiar with the child, and have ‘gone to much trouble to become accepted by the children of Midwest’ as (i) friendly, (ii) non-evaluating, and (iii) having an interest in what children do. The observers ‘have been trained to keep in the background’, trying to be ‘present but inconspicuous’. Another decision that aimed to diminish a possible observer’s influence was the choice of the age of the observed children: researchers assumed that at the age of seven, the ‘self-consciousness and social sensitivity are not great’ [9, p. 7]. Parents of the children were informed and enlisted as the researcher’s help, justifying the presence of the observers. They explained to the children that observers ‘would be interested in seeing what a seven-year-old boy did all day long’.

### (b) Recording contents and procedure

The observers were instructed to record everything the boy did. This included directly observable behaviour, vocalizations and movements but also their on-the-spot impressions and inferences of the boy’s perceptions, motives and feelings. Therefore, clearly, the humane sensitivity could be employed ‘to recreate for others the behavior of Raymond and the situations which confronted him as they experienced them’ [9, p. 8], where ‘others’ were presumably other scientists, ‘students of personality and social psychology’. The observers were selective, but ‘their intentions were to include as much as possible’ [9, p. 9].

The procedure states that each observational period lasted 30 min. Each observer had a board with a watch; the intervals for which the behaviours were noted were approximately 1 min. Observations were dictated into a sound recorder ‘immediately after they had been made’, which provided for spontaneous and full narrative in the presence of the listener who made notes upon unclear or ‘thin’ parts of the report. After the completion of the recording the listener interrogated the observer—these sessions were also recorded. The record was transcribed and edited by the observer, who deleted duplicates, enlarged meagre parts, corrected errors and ordered all incidents. Another staff member read the report and asked further clarifying questions; the answers to these questions were spliced into the record. Every observed action was presented as a single sentence, which was intended to preserve the ‘real units’ and enhance awareness of the time structure. Statements describing the physical or social situation to which Raymond responds are placed together in the same paragraph as the action description.

*One boy’s day* is the outcome of this procedure applied to 12 h in an ordinary day of Raymond. One of the goals of such research is to characterize the environment of a child in terms of places and situations in which their activities unfold, opening a possibility of quantifying differences in such settings and elicited behaviours for particular individuals [11]. Such rendering of the environment, in terms of socially constructed situations, is invaluable for our purposes of characterizing the life of a person after a laryngectomy. The clear methodology for establishing behaviour settings allows for detecting units of engagement in the social and physical world, which (i) may characterize the activity of a person before the laryngectomy and after; (ii) allow for comparisons between laryngectomees and people with an undisturbed voice in terms of differences in environmental engagements, possible avoidances of the situations, etc.; and (iii) allow for comparisons among individuals with laryngectomy to point to individual needs and problems. Our preliminary results point to vast differences in settings such as outdoor or indoor; involving many or few people; with intrinsic noise or without.

However, identification of the behaviour settings, while crucial, can be seen as only the first step in understanding the reasons for differences in engagements and in proposing ways and particular technologies to compensate. While Barker characterizes mostly non-interactive components of the settings, the recent focus on interactions in the cognitive sciences [12] makes it evident that characterizing behaviour settings is also a way of characterizing possible relations, encounters and engagements with people, whose interdependent actions are often vital parts of those settings, as Barker himself acknowledges [2]. It seems that Barker’s focus on predicting actions and on the maximal objectivity of the records made this particular property less prominent.

## 3. Diving deeper into interactants’ experience and microanalysis of interactions

Some work in ecological psychology itself has already sought to characterize modes of engagement and persons’ experiences within behaviour settings. Perhaps the most prominent acknowledgement of the fact that behaviour settings are constituted by social relations is the work of Harry Heft on ‘places’. Heft has developed a conceptual framework situating affordances within intentional actions [13], which naturally extends to understanding social engagement in such terms [14]. Places are structured environments supporting agents’ activities, often habitual ones and embedded in collective social practices, with shared collective intentions. Places are behaviour settings shaped by those intentions and development involves learning how to participate within these normatively constrained structures [14,15]. Even though such a richer characterization of the behaviour settings in terms of intentional actions is very helpful, it is important to point to the compatible approaches that propose concrete methodologies to study such intentional actions within social relations and experiences of people engaged in them.

In the realm of interactions of atypical speakers and their interlocutors, we argue that two specific issues need to be addressed. First, in deepening the understanding of complex, nuanced values to be realized in specific conversations (such as a gentle tone of voice expressing affection, or a boundary-creating harsh angry voice) we were looking for a method that would allow us to better understand the feelings, emotions and experiences of particular persons engaged in a given communicative encounter, as well as their relations to others in a particular setting. The same situation can be seen differently from a different person’s perspective; all the perspectives can be informative about how values are jointly realized, to guide the development of technology. Second, even in everyday interaction, realization of the desired expressions, emotions and modes of being together may reveal remarkable dynamic adjustment required by the given moment. This precision can be matched only by the microanalysis of movements, intonation, timing, gestures and other details of the interaction that might elude more coarse-grained analyses.

We claim that using the systematicity of Barker’s approach to the distinction of behaviour settings would allow for the creation of a catalogue of situations that need to be addressed with speech aids. By translating these observations into technological recommendations, we aim to stimulate the development of devices that not only facilitate communication but also are able to answer flexibly to various settings and kinds of participation, thus contributing to increased well-being. Aiming to encompass a wide variety of aspects relevant for participants in communicative encounters within a setting, we propose to complement Barker’s methodology with a framework for analysing intersubjective experience—engaged epistemology (e.g. in the work of Hanne De Jaegher [5]), and with conversational analysis suitable for in-depth study of linguistic interactions (Charles Goodwin’s approach [6]).

These conceptual and methodological frameworks broaden objective observations of interpersonal situations by using the subjective understandings of interaction by trained, and tuned, human observers. Both enactive approach and CA, even though coming from different backgrounds (psychology and anthropology, respectively), mark significant turns in the methodology of studying human interactions. This integration extends Barker’s original vision by acknowledging that human behaviour and development are not merely products of environmental forces but are deeply embedded in the dynamics of personal engagement and social interaction. Moreover, by focusing on the properties of a particular interaction as a unit of analysis, they allow delving into motives, feelings and emotions as shared experiences, as opposed to considering these characteristics as intrinsic properties of individuals or environment only. We claim that such a move, despite the risk of being less objective, offers a more nuanced understanding of human behaviour and experience. Furthermore, it will address Barker’s own concerns on researcher–participant relationship as visible from the quotes from *One boy’s day*: ‘When a geologist surveys and describes an area he does not change the geology of the region. But the presence of an observer of behavior often changes the psychological situation and hence the actions and feelings of the person observed. How can the student of psychological ecology keep the situation natural and observe naturally occurring behavior when it is not natural for an observer to be present?’ [9, pp. 5–6].

Here, Barker seems to openly acknowledge that persons are engaged in a number of relationships in a situation, which is natural. Why would a scientist’s interest in the life rhythms and social environment of a child be deemed unnatural? In fact, as described in §2, Barker selects and instructs the field workers based on this naturalness (familiar to a child, interested in children’s development) and enlists parents in research to help make the situation even more natural! Barker makes it clear that a subjective in-depth understanding of the subject’s social relations is part of defining and controlling the research procedure to be optimal for learning what we want to learn:

All who are concerned about the objectivity of this record must face two facts. One is that behavior without motives, feelings, and meanings is of little significance for students of personality and social psychology. The other is that motives, feelings, and meanings cannot be observed directly. In studying these problems it is necessary to work with the data available, however inadequate they may be. To those who are concerned about the bias and completeness of the record we can say only that, although the observers were inevitably selective in their perceptions of the rich and varied field of facts that Raymond presented, their intentions were to include as much as possible. To this it can be added that the use of a number of observers with differing unconscious biases and perceptual bents insured the record against any one person’s idiosyncrasies. [9, p. 9]

Engaged epistemology within an enactive approach addresses these problems, and we claim it is compatible with Barker’s ecobehavioural science. The enactive approach, beginning with the works of Francisco Varela, Evan Thompson and Eleanor Rosch, places a strong emphasis on first-person experiences and the active role of individuals in shaping their understanding and interaction with the world [16]. It is a position in cognitive science that argues that cognition arises through dynamic interaction between an acting organism and its environment.

Currently, the proponents of this perspective challenge the traditional cognitive science view of cognition as information processing by highlighting the centrality of human engagement and experience in cognition: *‘*Who is the expert when it comes to understanding people—the detached scientist or the ordinary person in everyday life?*’* [17, p. 5]. Following up on Vasu Reddy’s work [18], Hanne De Jaegher proposed ‘engaged or even engaging epistemology’ [5, p. 19] that will draw from human knowing, the approach to gaining knowledge from layman experience pertaining to everyday, remarkably important activities:

The drive towards understanding high intelligence ignores a wealth and a depth of human knowing that is right under our noses. Take knowing what is going on with someone from seeing the way they lift their gaze, how to build workable cities, how to read, write, and listen. Take knowing how to make maple syrup, knowing how to tame a fox, how to make ideas felt in poetry, music, or dancing, or how to playfully move between experientially different worlds. Take diagnosing psychiatric disorders, engaging with someone who is losing their mental capacities in dementia, dealing with breast cancer as a couple, or accompanying someone imprisoned to life on the outside. [5, p. 2]

In light of de Jaegher’s perspective, engagement between researchers and participants can be included among the natural phenomena from which valuable knowledge is obtained. Such an approach has led recently to the development of a method that seems to fill the need for enriching Barker’s behaviour settings and acknowledging the researcher–participant relationship. The method, PRISMA, is an approach that emphasizes engaging in relationships and drawing from the knowledge of lay people. It aligns well with our need to focus on the qualities of experience and interaction, which are otherwise elusive [19]. It is particularly effective in understanding complex social phenomena.

PRISMA has already been applied to understanding non-normative behaviour in autism, communication with people with dementia [5] as well as foetus–mother interaction [20], where typical forms of interaction may not be present or easily analysed by an external, detached observer. It explores the experiential perspective of individuals, offering insights into their unique ways of being and interacting with the world. This approach not only aids in comprehending atypical forms of interaction but also enriches our understanding of the diversity of human experience. This multitude of perspectives is reflected in the name of the framework itself, intended by its authors as an analogy to a prism that refracts light in its constituent spectral colours, allowing one to see the different aspects of the light while maintaining the essence of light itself. The authors apply this perspective for establishing goals to the understanding of the multiperspectival social interaction being at the same time a unit of analysis as a whole and the conglomerate of experiences of individuals.

PRISMA is organized in the form of a workshop with a systematic protocol for investigating interaction experience based on an embodied methodology and concepts. Participants are provided with templates for noting their observations regarding their own experiences. The workshop involves a group of people in a particular interaction we are interested in, such as joint exercise, playing music together or dancing. Participants are instructed on how to report experiences in a systematic way and are provided with a predefined time frame of interactions as well as a predefined matrix of focus areas on which they are writing down their insights. The interactants are instructed to focus on their respective experience and on its specific aspects in each experience interval; the areas of focus are sensing (basic sensual experience), feeling (their emotions) and thinking (mental operations). Participants in a PRISMA experiment (not understood as an event studied in the laboratory, but the type of practice) are encouraged to use their own intuitions as they are considered the best measuring devices that sense, feel and think about their and others' experiences. Firstly, participants observing experience are focusing on self-perception (e.g. ‘what I feel’ in the ‘feeling phase’), then on other-perception (e.g. what the other person feels), and finally on *in between* (what we feel together). This allows capturing various aspects of experience happening between two interacting people. PRISMA can complement the fieldwork (observing naturally occurring behaviour) and can be used in arranged interactive situations that resemble the ones observed in nature (or even in specified intervals of a natural event).

Nevertheless, however, deeply PRISMA delves into the experiences of participants, it does not involve detailed interaction analysis; experiences reported often pertain to a whole interval of interaction and not to specific moves and events. Communication analysis requires a fine-grained study of movement, gaze and voice coordination capable of detecting moves, glitches and repairs in conversation, which could escape conscious experience yet be important factors in evoking certain experiences. To capture these details of interaction on a fine-grained timescale, we need a perspective complementary to the enactive approach.

While enactivists are focused on particular perspectives of people engaged within an interaction, CA researchers focus on a detailed analysis of a particular sequence of turn in a conversation and particular vocalizations, bodily movements and participants' inferences about motives and feelings underlying these actions. CA brings to light the intricate dynamics unfolding between co-acting people. Moreover, the work of Charles Goodwin, one of the principal researchers within the approach, illustrates the potential of CA to uncover the subtle ways in which personal experiences and social interactions intertwine, providing a deeper understanding of the co-constitutive nature of communication [6].

The methodology of CA employs a particular tool for discovering such nuances: video-capturing, meticulous transcription and rich linguistic analysis of interactions. The special description of ‘language’ of video-recorded interaction allows for a comprehensive examination of the subtleties of human communication. Through detailed microanalysis (on a timescale of fractions of a second), researchers can unravel complexities of conversational dynamics, including timing, prosody and the use of gestures, thereby gaining a deeper insight into how meaning and understanding are co-constructed in social interactions. Turning to linguistic analyses, researchers, through the lenses of CA, become aware of the complex use of language and its structures and acknowledge that a particular ‘meaning’ is always created in concert with others in a meaningful (verbal or not) interaction.

An example of such interaction nuances that can be clearly observed in their full range are conversations of a speaker with aphasia who was able to produce only the words ‘yes’, ‘no’ and ‘and’, while still being an active speaker in the family conversations, ‘a focal point’ [21]. Privately he was Chuck Goodwin’s father, and after several years of informal observation of how rich his ‘language’ was (comprising rich intonation, gestures and reusing linguistic resources produced by others), Goodwin decided to video-record his father’s interactions. The video data used for further linguistic analysis include transcripts, video frames and spectrograms. The meticulous transcription and attention to gestures and voice pitch revealed that the particular ‘yes’ and ‘no’ of a person meant something different depending on the immediate context. The analysis of such specific interaction details also needed engagement with the video data and the use of human knowledge for analysis of these particular social cues by linguists skilful in video analysis. This allows us to reason about the structure of interaction and the agency realized by particular individuals.

The effort to be objective and adhere to a quantitative description of the environments and actions generates tension because observers are humans who remain in relations with the observed. This is, indeed, noted by Barker: ‘any interaction of Raymond with an observer is real behaviour with significance in its own right. Every such interaction can be accepted as telling something about Raymond as a particular boy of Midwest.’ However, Barker’s and Wright’s record does not seem to facilitate fleshing out this ‘something’. It seems that the role of researchers as ‘sensors and transducers’ obscures their agency as modifiers of the situations, i.e. causes of events in which valuable data can be gathered. Therefore, even though the pursuit of the ‘psychologist-free units’ of behaviour as opposed to ones carefully altered by a ‘psychologist as operator’ in a laboratory is fully commendable, the impossibility of removing a ‘psychologist as a human being’ from the picture is clear even from this very record. In the next section we provide examples from *One boy’s day* and our research that shows the need for elaboration on Barker’s framework.

## 4. Interactions of persons within behaviour settings

An illustration of the agentive role of the observers and the necessity of drawing more attention to complex human relations, engagements and experiences in culturally and situationally moulded interactive routines, consider the following excerpt from Barker’s group record of Raymond’s dressing up for school [9]:

7:00

(…)

He sat up and rubbed his eyes.

He glanced at me and smiled.

I smiled in return as I continued making notes.

Mrs. Birch took some clothes from the bureau and laid them on the bed next to Raymond. There were a clean pair of socks, a clean pair of shorts, a white T-shirt and a striped T-shirt. Raymond’s blue-jean pants were on a chair near the bed. Mrs. Birch continued to stand beside the bed.

7:01. Raymond picked up a sock and began tugging and pulling it on his left foot.

As his mother watched him she said kiddingly, ‘Can’t you get your peepers open?’

Raymond stopped putting on his sock long enough to rub his eyes again. He appeared to be very sleepy.

He said plaintively, ‘Mommie,’ and continued mumbling in an unintelligible way something about his undershirt.

7:02. His mother asked, ‘Do you want to put this undershirt on or do you want to wear the one you have on?’

Raymond sleepily muttered something in reply.

His mother left the room and went into the kitchen. Raymond struggled out of the T-shirt which he had on. He put on the clean striped T-shirt more efficiently.

7.03. [7.03 am – red.]

He pulled on his right sock

He picked up his left tennis shoe and put it on.

He laced his left shoe with slow deliberation, looking, intently at the shoe as he worked steadily until he had it all laced

7.04. He put on his right shoe.

He laced up his right shoe. Again he worked intently, looking at the shoe as he laced it.

His mother called, ‘Raymond, do you want an egg for breakfast’ in a pleasant, inquiring tone

Raymond responded very sleepily, ‘No.’ His voice showed no irritation or resentment, he just answered in a matter-of-fact, sleepy way, ‘No.”’

7.05. As he finished lacing his shoe, he called out in a rather plaintive voice, ‘Mommie, come here.’

Mrs Birch didn’t respond verbally, but her footsteps signalled her approach

*When his mother came into the room, Raymond still had on his pajama pants; his shorts were lying on the bed next to him*

Mrs Birch came over to the bed and bent down close to Raymond.

He whispered something to her

Mrs. Birch chuckled with slight embarrassment and said laughingly, ‘Well, take them off and put them on,’ meaning that he was to take off his pajama pants and put on his underwear pants.

She stood next to him as he made the change.

*Mrs Birch returned to the kitchen*

7.06. Raymond put on his blue-jean pants as he stood by his bed.

Honey, Raymond’s fat, broad, elderly fox terrier, ambled into the room.

Raymond greeted her in a sleepy but friendly voice ‘Hi, Honey.’

Honey put her front paws on Raymond’s knees.

He scratched her back and patted her as he finished buckling his belt.

It is not a common behaviour to put on one’s shoes before taking off pyjama pants, and if the result of the study was just a count and sequencing of behaviours in different settings, this would certainly be an odd-one-out. Only if one imagines what the presence of a stranger can do to a 7-year-old boy who needs to undress from his pyjama bottoms in order to put on his underpants, the sequence and the intervention of the mother begin to make sense. The short and matter-of-fact listings of the actions do not accurately account for the boy’s possible experiences and the layers of social engagement which define the situation. The interactive behaviours (‘he whispered something to her’ and ‘she stood next to him when he made the change’) are described on a par with other behaviours (e.g. ‘he laced up his right shoe’), so the feelings (probably of embarrassment and perhaps relief) and the values realized in interaction (decency in front of the stranger, reassurance from the mother) elude the reader. They are never commented on, which is detrimental even to the record itself: we will never know if the boy took off his shoes and put them on again—it looks like these actions were omitted from the record. Such complex layers of engagement [21–23] are missing from the record, while they are crucial for understanding people’s experiences and thus the understanding of causes for actions and participants’ well-being within behaviour settings.

The conscious choice of the group not to start the investigation with recognition of the observers' perspective attenuated the observer’s influence (e.g. by selecting eight observers that changed during the boy’s day). This was to increase the probability of accurately recognizing the structure of the environment. This choice designated the path for methodology development that was already biased (the extensive role of the physical environment and engaged actors neglected the role of the observers). The individual contribution of observers, their feelings and their understanding of motives were not so important. However, even in the earliest foundational work, the Kansas Group understood that such nuanced factors could be inferred by the observers, making behaviour more understandable by other scientists, but those intuitions, it seems, were not confirmed directly by the observed participants.

On the contrary, our initial problematic situations (see figure 1) were observed by us from first- and second-person perspectives of interactants. One of them was ‘Singing with the guitar by a campfire on an integration trip with a new group of friends’. In this situation, speech and gestures play along together. Such parties often include eating and drinking together, which is also problematic for people with speech and vocal tract impediments, or dynamically joking with each other. In such situations, timing (on a timeframe of milliseconds) is crucial; the note in a song or a joke does not ‘play’ well when delayed or when it needs to be repeated (which happens frequently for people without a larynx). Enriching ecobehavioural science with CA and engaged epistemology allows us to dive deeper into situations possibly at the heart of someone’s well-being. Singing by the fireplace requires speaking loudly, which can be provided by a voice amplification device. Playing the guitar at the same time is only possible when a person can use both hands (which can be provided by a design of a hands-off device contrary to traditional voice restoration devices for laryngectomees). A person’s voice augmented with a speaker can be more appropriate for the given setting (windy environment, sound that should be understandable for all regardless of the direction where they are located in relative position to the speaker). All of those aspects require clear indications of what would make a person feel better (because they told us so), and how technological solutions can facilitate that.

A similar pattern was observed by us in our ethnographic field research with atypical speakers. For example, an entrepreneur without a larynx, before the surgery, had been using his voice to talk harshly over the telephone with other people who had not paid him overdue invoices. This reveals his need for convincing somebody of his opinion in such business phone calls, where the voice was a tool used in a specific social relation, and this particular need to be satisfied required a voice which is used with timely adjustment and the adequate timbre. This particular person uses an electrolarynx and amplification and is willing to improve his communication with new devices. The same quality can be generalized to any conversation with someone who is reluctant to speak with us. It illustrates the feelings, goals and values realized in the interaction (e.g. the need to change someone’s opinion, or express compassion or empathetic support). Sufficient interaction quality in the design of technology will be needed to accommodate such nuanced communicative requirements.

Our method allowed us to understand such situations that occur rarely, and the fact that they are rare is precisely the problem. What we are interested in is not only the quantity of interactions, but *how* exactly these interactions differ. This example illustrates how important particular values are, demonstrated in specific micromovements, dynamic pitch changes, gestures and crucially, the timing of their realization. It also, similarly to Barker’s ‘Pyjama Example’, emphasizes the importance of specific qualities pertaining to relationships and values expressed in micro-movements and personal experiences, rather than merely focusing on voice intelligibility—even in a typical ‘phone conversation’ example we observe very specific needs.

If we conceive of situations as crucially created by participating persons, we can enrich Barker’s notion of behaviour settings by openly acknowledging this fact. Perhaps the way to integrate both persons and their relations, and the role of environment in the analysis is to allow (i) for the relations to play a more central role in the record, acknowledging that they co-construct persons in settings [24] and (ii) give a voice to the experiences of the people involved, who are not visible in the original Barker’s record. This calls for methods that are sensitive enough to recognize the relations without destroying the setting descriptions and allow for capturing experiences, without destroying engagements in situations. To summarize, the ‘settings’ are made of places, persons, relations and artefacts involved. These are ‘eco-behavioural structure[s] wholly new in the research literature’ [25]. Participation in those settings [8] is observable and even countable, which is a crucial point in our quest for characterization of a situation and a person’s needs. It allows us to ask a vital question: what are the ‘settings’ that we are looking for in our particular research on laryngectomee interaction? However, a deeper characterization of the setting would involve recognizing both the types of engagement in interactions with people comprising a setting and individual experiences within those engagements. It is needed both for the understanding of the setting from different viewpoints and for understanding which changes might be influenced by deliberate design.

## 5. Conclusions and next steps

Barker’s behaviour settings theory gives us hope that meaningful units of analysis can be identified and used for the study of communication of people after laryngectomy. This is crucial in order to characterize the specific needs and requirements settings pose to persons with atypical speech and to assess which kinds of devices can alleviate the problems encountered. However, we also note that the methodology embraced by Barker can be augmented by new developments in the study of interaction and engaged epistemology.

Rapid progress in artificial intelligence and new materials will likely allow for the design of devices better suited to alleviate major voice impairment problems. We claim that these new emerging technologies should be in closer contact with novel psychological research, which recognizes the structure of the environment and human interactivity in the given setting. Barker’s framework is useful for this goal, because it allows us to classify situations which are standing patterns of behaviour in particular physical situations. The designers and engineers need such research tools to adequately catalogue the situations for which they are developing the technology, as ways to study extra-individual units of behaviour.

However, each instantiation of human–human interaction reveals diverse communicative needs and qualities of the voice required for fulfilling them. Finding solutions for specific contexts important for a given person is crucial for successful communication and thus can alleviate their well-being on a longer timescale. The need for physical aspects such as an intelligible voice can be derived from recognition of behaviour setting only, but the specific qualities of the voice, such as a voice which is not gentle enough, comes from interactive, nuanced aspects of conversation.

Both methodologies proposed in this article as behaviour setting enrichment prioritize activity in the world and engagement with others as a main motivation for cognition and communication [26]. The enactive approach emphasizes the engagement needed to understand human-interactive phenomena and linguistic anthropology, with its CA methodology, supplies valuable tools for relation-centred research and analyses, including in-depth video analysis of crucial interaction details. These two research traditions, while being developed separately from Barker’s work and from each other, do complement each other, allowing for zooming in and zooming out on particular aspects of social interactions. Such enrichments do not question Barker’s approach but rather fill in the information left unstudied. Complementing behaviour settings by experience sampling, the acknowledgement of self and others’ perspectives and the in-depth analysis of video-recorded interactions would facilitate understanding the shape of the setting and particular experiences.

We claim that incorporating specific elements of these methodologies into the behaviour settings framework may lead to the development of a nuanced, humane or ‘tender’, research approach that acknowledges and preserves the crucial values manifested in relationships and in particular situations. This, in turn, should facilitate the development of technological specifications designed to allow their users to influence interactions in alignment with these identified values. Recognition of microscale movements in dyads, and in particular human–human relationships, can reveal what *kinds of control* in speech intonation, timings and gestures are needed to realize particular values. These capabilities can then be enabled, enhanced or constrained by speech aid technologies.

Barker’s approach can also help us decide which carefully chosen elements of the natural settings could be brought into the laboratory situation, preserving most of the physical and social structure engaging the participants, and which are best studied in natural observation or engagement of researchers in natural settings. Such research practice is based on the awareness that a psychological lab is not ‘a situation without qualities’ that renders results generalizable to all other situations, but rather a carefully transplanted tissue of the world on which natural behaviours might appear, thrive and be studied. Barker’s research framework can be enriched to encompass the kind of engagements that are essential in the study of the human–machine coordination problem within human–human relationships [27].

Some insights in our research were possible only because of the active participation of people from the affected community and our own engagement with them. We drew from our own first and second-person experience as interacting humans, not only as researchers. Our next steps in the implementation of this integrated research methodology is to engage with speech therapists, other medical professionals and speech aid designers to confront and integrate the methodologies presented in the article with their own workflow and insights. Only then can specific recommendations be made regarding technology development processes and outcomes based on the empirical data from the field. Roger G. Barker’s ecobehavioural science was extremely useful in this endeavour as it has provided us with the concepts, methods and categories of dividing the stream of behaviour into distinguishable units and this is the thought that we aim to bring along with the development of a comprehensive framework for speech aid design.

## Data Availability

This article has no additional data.
